# Pennsylvania: Appeal for Greater Interest in the U. S. V. M. A.

**Published:** 1898-03

**Authors:** W. H. Ridge

**Affiliations:** Resident State Secretary; Trevose, Bucks Co., Pa.


Secretary.

### PENNSYLVANIA—APPEAL FOR GREATER INTEREST IN THE U. S. V. M. A.

We wish to call your attention to the fact that it is the duty of all qualified veterinarians to join the United States Veterinary Medical Association.

1st. It is an honor to belong to the highest Association in this country, and all veterinarians should have a desire to add their names to its roll; it gives to them a standing that justly belongs to every good veterinarian.

2d. It is a source of education that cannot be received by any other method. We get the education of many minds concentrated and meted out to us in a manner that could not be appreciated by reading or, in fact, by any other way than orally.

3d. We should understand what will do the most good to the greatest number in our profession; by uniting we can do this; by individual action we cannot. To quote Professor Hamilton, in speaking of the Farmers' Institute, he says: "The great weakness of our country people lies in their lack of consolidation of thought upon a given question.

"When all of the country people agree upon any given subject, their desires will be gratified. Their failure lies in their lack of agreement, and the lack of agreement is usually due to a lack of accurate knowledge of the subject and its true bearing upon their industry."

This, we think, is applicable to us as a profession. While local associations are good educators, the meeting of the whole body of veterinarians can consolidate thought upon questions that are of vital importance to us as American veterinarians, that cannot be done by local or other associations.

4th. The mercenary view—take it in a business light; will it pay? Yes, it will pay, even if you never attend any meetings. You will receive the printed proceedings of the Association, and in numerous other ways receive more than your money's worth.

While other occupations returning a revenue equal to the veterinarian's are often taxed one hundred or more dollars per year, the veterinarians are lightly taxed. Surely, then, we should deem it our duty to self-impose a tax to help our Association, which is ourselves, even if we cannot make ourselves believe it an imperative duty to attend the meetings. If we could induce every veterinarian in this country to join and take an active interest in this Association, we would advance professionally in the next ten years far beyond the belief of the most imaginative mind among us. Thus by elevating the profession we elevate ourselves, and by elevating ourselves we gain larger and better practice, proving it to be a paying investment to join this Association.

We hope that we have made it clear to you that it is your duty to join this Association. If you will send me your cheque for five dollars I will forward the same to the proper officers; your name will then be presented at the next meeting, September 6, 7, and 8, 1898, at Omaha.

We also desire you to send us reports of the prevailing diseases in your neighborhood, with any other items that will be of interest to the Committee on Diseases.

Now, we have tried to convince you that it is to your interest to join this Association. Will you favor us by a reply by return mail?

W. H. Ridge,
Resident State Secretary.

Trevose, Bucks Co., Pa.

